# Homing and Nest Recognition in Nocturnal Blue Petrels: What Scent May Attract Birds to their Burrows?

**DOI:** 10.1007/s10886-023-01424-3

**Published:** 2023-05-26

**Authors:** Timothée Zidat, Marianne Gabirot, Francesco Bonadonna, Carsten T. Müller

**Affiliations:** 1 School of Biosciences, Cardiff University, Sir Martin Evans Building, Museum Avenue, CF10 3AX Cardiff, UK; 2 UMR Physiologie de la Reproduction et des Comportements, INRAE, CNRS, Université de Tours, IFCE, Nouzilly, France; 3 ADENA - Réserve Naturelle Nationale du Bagnas, Domaine du Grand Clavelet, Route de Sète, 34300 Agde, France; 4 CEFE, Univ Montpellier, CNRS, EPHE, IRD, Montpellier, France

**Keywords:** Homing Behavior, Nest Air Odor, Olfaction, Orientation, Procellariform Seabirds, TD-GC-TOF-MS

## Abstract

Hypogean petrels return to the same nest burrow to breed on remote islands during the summer months. Their nocturnal behavior at the colony, strong musky odor and olfactory anatomy suggest an important role of olfaction in homing behavior and nest recognition. Behavioral experiments showed that olfactory cues are sufficient to allow nest identification, suggesting a stabile chemical signature emanating from burrows and facilitating nest recognition. However, the chemical nature and sources of this odor remain unknown. To better understand the nest odor composition, we analyzed volatile organic compounds (VOCs) of nests of blue petrels (*Halobaena caerulea*) derived from three different odor sources: nest air, nest material and feather samples. We also compared, during two successive years, VOCs from burrows with an incubating breeder on the nest, and burrows used during the breeding season by blue petrels but shortly temporally unoccupied by breeders. We found that the nest air odor was mainly formed by the owners’ odor, which provided an individual chemical label for nests that appeared stabile over the breeding season. These findings, together with the previous homing behavioral studies showing an essential role of the sense of smell in blue petrels, strongly suggest that the scent emanating from burrows of blue petrels provides the information that facilitates nest recognition and homing.

**Supplementary Information:**

The online version contains supplementary material available at 10.1007/s10886-023-01424-3.

## Introduction

Procellariforms (petrels, albatrosses and shearwaters) have a well-developed sense of smell, particularly important for orientation in the open sea (Bonadonna et al. 2003a; Gagliardo et al. 2013; Bonadonna and Gagliardo 2021). Behavioral experiments in several burrow-nesting species also demonstrate that olfactory cues are important in short-range homing and nest recognition (Bonadonna and Bretagnolle 2002; Bonadonna 2009). For example, experimental displacement of the burrow entrance of blue petrels does not affect their capacity to find their nest suggesting that birds use olfactory cues and not positional cues (Bonadonna et al. 2004). Additionally, binary choice experiments show that petrels, including blue petrels, are able to distinguish the odor of their own nest from that of a conspecific solely relying on olfactory cues (Grubb 1974; De León et al. 2003; Bonadonna et al. 2003b, c, 2004; Jouventin et al. 2007; O’Dwyer et al. 2008; O’Dwyer and Nevitt 2009). However, despite the behavioral evidence for the role of smell in nest finding behavior of petrels, the chemical nature, composition and origin of olfactory cues emanating from the nests remain unclear (Bonadonna et al. 2003c; Mardon et al. 2010).

Blue petrels (*Halobaena caerulea*) are pelagic birds which live most of the time in the open sea in the Southern Ocean and breed annually with the same partner in the same burrow on small oceanic islands around Antarctica (Warham 1990). They nest underground in dense colonies, and each pair lays a single egg per year. The partners take turns to incubate it relieving each other from the nest every 8–12 days during approximately 45–50 days of incubation (Chaurand and Weimerskirch 1994). At the start of the breeding season, blue petrels return to the same nest after a year-long absence. They approach their nest at night, often when it is completely dark, avoiding moonlit nights, to elude predation risks from other birds such as skuas and gulls (Warham 1990; Healy and Guilford 1990; Mougeot and Bretagnolle 2000; Bonadonna and Bretagnolle 2002). However, as petrels have no nocturnal adaptive vision (Brooke 1989; Warham 1990, 1996; Martin and Brooke 1991), nest recognition is based on other cues such as olfactory cues.

Blue petrels incubate their single egg alternately, one partner leaves the nest at a time to forage at sea and then comes back to relieve its mate (Warham 1990). Most of the time, returning birds find their incubating partner but sometimes the shifts are not perfectly synchronized, leaving the egg alone for days, which, however, does not pose any danger to the developing embryo inside the egg (Chaurand and Weimerskirch 1994). In this case and during the chick-rearing period, where partners also take turns feeding their chick, the returning partner has to recognize an empty nest. It is, therefore, likely that nest recognition is derived from a mixture of both owners’ odors with probably a bigger contribution from the latest partner (Bonadonna et al. 2003b, 2004; Mardon and Bonadonna 2009). Indeed, recent chemical analyses show that the odor of petrels (presumably from uropygial secretion and/or feathers) contains information about the bird’s identity (Bonadonna et al. 2007; Mardon et al. 2010, 2011; Jennings and Ebeler 2020), and binary choice experiments demonstrate that petrels are able to discriminate and recognize their mate (Bonadonna and Nevitt 2004; Jouventin et al. 2007; Mardon and Bonadonna 2009), as well as their own odor (Bonadonna and Nevitt 2004; Mardon and Bonadonna 2009). In addition, nest olfactory cues may also come from material that the birds use to make their nest in the incubating chamber (at least in Kerguelen area; personal observation). This material mostly consists of the owners’ feathers, dead branches and roots of *Acaena magellanica* and few other alien plants (e.g., *Tarassacum* spp.; Chapuis et al. 2004). Finally, the soil may add a common odor to all the nests. Consequently, all of these odor sources might contribute to the nest signature used by birds in nest recognition during the breeding season and when returning after a year’s absence.

To better understand the nest recognition mechanism in blue petrels, we collected olfactory cues (VOCs) from two different odor sources (nest air and nest material samples) from nests occupied by blue petrel breeding pairs, as well as feather samples from the incubating birds. This was done on Ile Verte, a small Island in the Kerguelen Archipelago, over two seasons (2014 and 2015). We hypothesize that (i) each nest caries specific odor, which is stable over time, especially during the breeding season; consequently we expect (ii) no major differences between nest air samples from occupied (with a bird in the nest) and empty nests (without a bird at the time of collection but not abandoned). We also hypothesize that (iii) nest air, nest material and feather samples have different chemical compositions, and (iv) nest air samples consist mostly of compounds from bird’s odor as part of the olfactory nest label.

## Materials and Methods

### *Study Period and Location*

All samples were collected during two successive austral summers (from November to January 2014–2015 and 2015–2016; Tab. S1) as part of the French Sub-Antarctic program ETHOTAAF n°354, on Ile Verte (49° 51’ S, 70° 05’ E) in the Kerguelen Archipelago in the Southern Indian Ocean. This small island of approximatively 2 km^2^ is a breeding site for many burrow-nesting petrels including our subject species, the blue petrel (*Halobaena caerulea*). To make the reading easier, the name of the sampling year will be shortened to 2014 and 2015, respectively.

### *In Situ Odor Collection*

To characterize the volatile organic compounds (VOCs) emanating from nests of blue petrels, we collected three different types of odors in the field: nest air, nest material and feather samples (summarized in Tab. S1). We sampled (a) nest air as a proxy for the olfactory cues emitted from burrows with a breeder in the nest (hereafter called in the manuscript “occupied nest”) and burrows used during the breeding season by blue petrels but shortly temporally unoccupied by breeders (hereafter called in the manuscript “empty nest”), (b) nest material from some of these nests as an alternative access to nest odors, allowing characterization of chemical compounds from material constituting burrows (e.g., plants, roots, soil) and (c) feathers of birds nesting in some of these nests as a proxy for bird’s odor as well. We considered nest air samples as a proxy of the scent emitted from burrows and enabling nest recognition by the owners. By collecting these three different odor sources, we expected to be able to decode the chemical composition and the origin of the olfactory cues emitted from nests.

#### *Nest Air Samples*

**Fig. 1:**
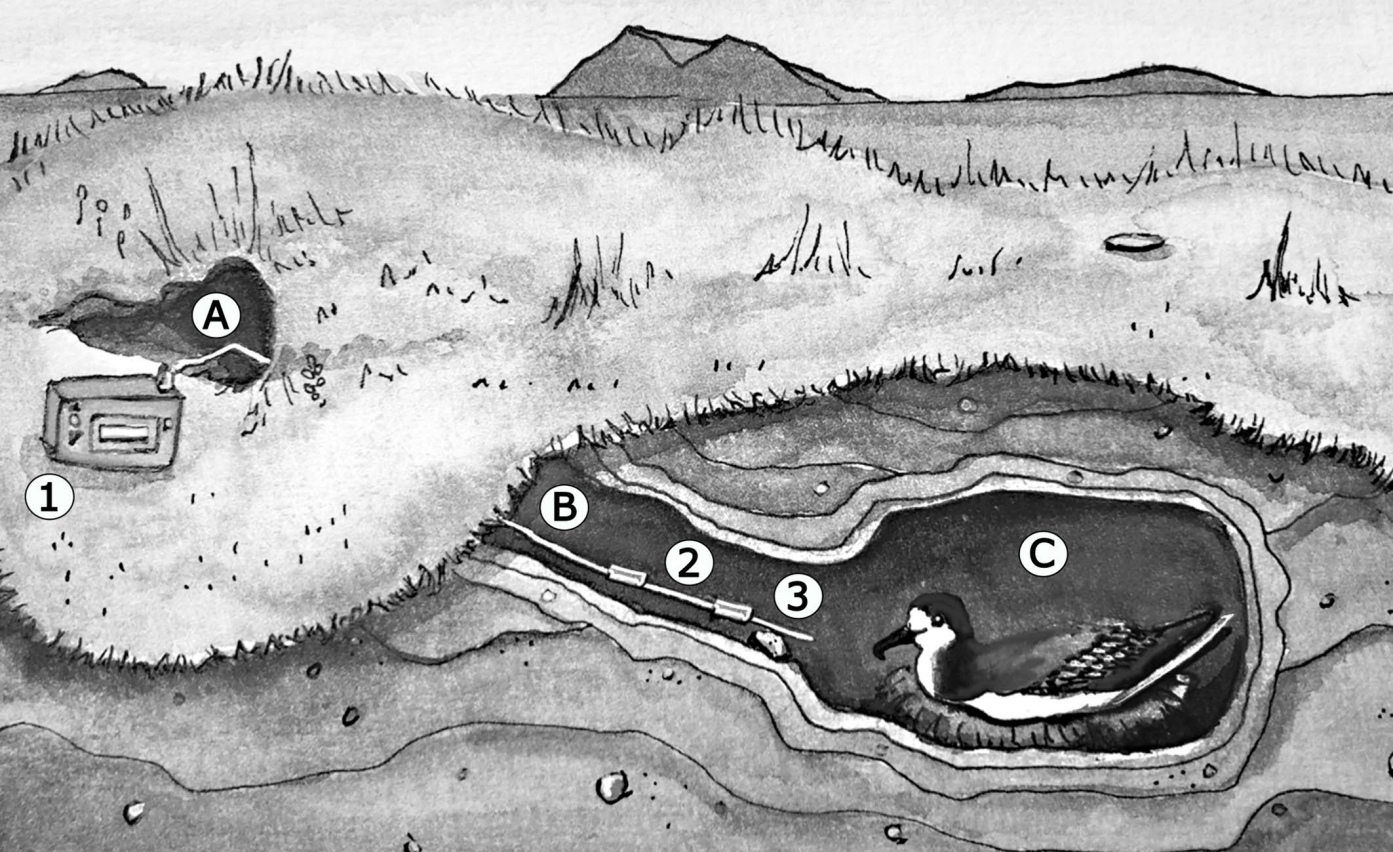
Experimental set-up for nest air sample collection with an incubating bird. A: nest entrance, B: access tunnel, C: nest chamber, 1: pump, 2: SulfiCarb tube and 3: Tenax® TA tube (Drawing done by Élodie TEXIER)

VOCs from nests of blue petrels were collected *in situ* (Fig. 1) from 15 nests during two field seasons (2014 and 2015, Tab. S1). Two tubes, connected in sequence, were placed in the nest chambers, the first tube was packed with Tenax® TA adsorbent (150 mg, Markes International, Llantrisant, UK) and the second with SulfiCarb (150 mg, Markes International, Llantrisant, UK). Nest air was sampled for 3 h at a flow rate of 50 mL/min using a GilAir™ PLUS personal air sampling pump (Gilian®). Samples were collected from nests with an adult incubating inside (N = 16 in 2014 and N = 12 in 2015), and from empty nests (N = 3 in 2014 and N = 5 in 2015). Empty nests were sampled in cases where shifts were not perfectly synchronized and nests were empty for 1–3 days. In two cases (Nest 17 in 2014 and Nest 29 in 2015) one of the birds failed to return (Tab. S1). Replicate sampling of the same nest was undertaken when possible (Tab. S1). All tubes were capped after collection and stored at ambient temperature (about 4 °C) until laboratory analysis. To control for potential contamination during storage and/or transport from the island to the laboratory, five control tubes (closed tubes traveling to the field station and back without being used) were added.

#### *Nest Material Samples*

Nest material (N = 14) were collected from eight nests during the 2014 field season, from nests with an adult incubating inside (N = 12), and for two of them from an empty nest (see Tab. S1 for details). Approximately 100 g of nest material (soil, plants, roots, feathers) were placed into 125 mL opaque glass jars (Fisherbrand^TM^, amber straight sided round bottle) wearing clean nitrile gloves. Four empty vials were included as controls for potential contamination during storage and/or transport. All samples were stored at ambient temperature (4 °C) in the field and then at -20 °C until laboratory analysis.

#### *Feather Samples*

Feathers were collected from 10 birds during the 2014 field season. Because Kerguelen is subject to very strong winds, typical of these latitudes, feathers were collected after birds were removed from their burrow and transported in an opaque cotton bag to the field laboratory established on the island (less than 80 m away from where birds were captured). Also, all the manipulations or odor collections were performed before 4pm, to allow birds to calm down before nightfall around 10pm. Around 100 mg of feathers were cut from the bird’s down, packed in nalophan® then wrapped in aluminium foil and placed in an individual sealed plastic bag. Samples were stored at 4 °C during the 2–3 weeks in the field and then at -20 °C until laboratory analysis.

### *Odor Analyses*

#### *Nest Air VOCs*

Tubes were analyzed at Cardiff University (Wales, UK) using a thermal desorption gas chromatography time-of-flight mass spectrometry (TD-GC-TOF-MS). All samples were spiked with 0.5 µL of 50 µg/mL biphenyl in methanol (Sigma-Aldrich®, France) as an internal standard (= 25 ng per TD tube). Primary desorption was carried out by a TD100 thermal desorption system (Markes International Ltd., Llantrisant, Wales, UK) at 280 °C for 10 min with a flow rate at 40 mL/min. Analytes were re-collected on the Tenax® TA trap at 25 °C, desorbed at 250 °C with a helium split flow of 5 mL/min for 1 min and injected into the GC (7890A; Agilent Technologies, Inc., Didcot, UK) using a helium flow of 2 mL/min resulting in a split ratio of 3.5:1. Samples were separated over a 60 m, 0.32 mm ID, 0.5 μm R x 5 MS capillary column (Restek, Bellefonte, PA, USA) using the following temperature program: start at 35 °C for 0.5 min, then 8 °C/min to 100 °C, then 5 °C/min to 280 °C, and 10 min hold at end. Mass spectra of compounds from EI at 70 eV were recorded over the mass range from m/z 30 to 350 using a time-of-flight mass spectrometer (BenchTOF-dx, Almsco International, Blue Ash, OH, USA) with transfer line temperature of 250 °C and the ion source temperature at 200 °C. A mixture of C_8_-C_20_ alkanes (1 µL, 40 mg/L each alkane, Sigma Aldrich®, Switzerland) was injected into a blank tube and processed under the same conditions to calibrate for retention index (RI) calculation. Preliminary analysis showed that no chemical compounds were recovered from SulfiCarb tubes. Thus, subsequent analysis used only chemical profiles from collection on Tenax® TA tubes. All tubes were conditioned at Cardiff University before each sampling campaigns using a program at 320 °C for 60 min (in 2014) or 30 min (in 2015) with a helium flow rate at 20 mL/min, and analyzed to control for the cleaning efficiency.

#### *Nest Material VOCs*

In the laboratory, VOCs were extracted directly from the headspace of the sample jar with solid-phase microextraction (SPME). VOCs were collected onto a 2 cm DVB/CAR/PDMS composite fiber (50/30 µm divinylbenzene–carboxen–polydimethylsiloxane, Supelco) for 1 h at 60 °C in a water bath. Following collection, fibers were immediately desorbed for 2 min at 250 °C in the injection port of the GC-MS instrument (GC 6890 N, MSD 5973 N, Agilent). VOCs were separated on 30 m, 0.25 mm ID, 0.25 μm Zebron 5 MS capillary column (Phenomenex, Macclesfield, UK) using the following temperature program: 35 °C for 0.5 min, then 8 °C/min to 100 °C, then 5 °C/min to 280 °C and 10 min hold. The interface temperature to MS was held at 250 °C and the ion source temperature at 200 °C. The MS was used in scan mode over the mass range from m/z 35 to 350 and in EI with 70 eV. A mixture of C_8_-C_20_ alkanes (0.1 µL, 40 mg/L each alkane, Sigma Aldrich®, Switzerland) was injected directly and processed under the same conditions to calibrate for RI calculation.

#### *Feather VOCs*

In the laboratory, 44–48 mg of feathers from each sample were placed into a ‘Loose Fit’ Teflon® insert (Liner PTFE; Markes International Ltd., Llantrisant, Wales, UK) which was inserted into a clean empty TD stainless steel tube (OD = 6 mm; L = 88 mm; Perkin-Elmer France, Courtaboeuf, France). A silanized glass wool plug (Perkin Elmer USA) was added on the top of the tube to avoid any loss of feathers. Empty tubes (without feathers) were added to each run and used as control. All samples were spiked with 0.5 µL of an internal standard solution (biphenyl solution diluted at 50 µg/mL in methanol; 25 ng injected in each TD tube; Sigma-Aldrich®, France). VOCs were extracted from feathers using direct thermal desorption (TD), the process of heating samples in a flow of nitrogen, an inert gas like helium. Analytes were re-trapped on a secondary adsorbent trap and injected into the GC-TOF-MS system. Although extraction efficiency of thermal desorption is lower than that of solvent extraction (Baltussen et al. 2002), the absence of a solvent dilution effect generally makes it more sensitive overall. The combustion point of feathers is around 230 °C (J. Mardon personal data), thus, we tested different temperatures (70, 100 and 180 °C) of desorption to choose the optimal one. We saw some modifications in chemical compounds and excessive desorption of waxes at elevated temperatures (M. Gabirot and C.T. Müller personal data). Tubes were thus desorbed at 100 °C using a TD100 thermal desorption system (Markes International Ltd., Llantrisant, Wales, UK) for 20 min, and the trap desorbed at 300 °C for 1 min, with a helium split flow of 5 mL/min into the GC using a helium flow of 2 mL/min. This resulted in a split ratio of 3.5:1. VOCs were separated over a 60 m, 0.32 mm ID, 0.5 μm R x 5 MS capillary column (Restek, High Wycombe, UK) with 2 mL/min helium as carrier gas under constant flow conditions using the following temperature program: initial temperature 35 °C for 0.5 min, 8 °C/min to 100 °C, then 5 °C/min to 280 °C, final hold for 10 min. The interface temperature to MS was held at 250 °C and the ion source temperature at 200 °C. Mass spectra were recorded from m/z 30 to 350 on a time-of-flight mass spectrometer (BenchTOF-dx, Markes International Ltd, Llantrisant, UK). A mixture of C_8_-C_20_ alkanes (Sigma Aldrich®, Switzerland) was processed under the same conditions to calibrate for RI calculations.

### *Chromatographic Analysis and Data Pre-treatment*

Chromatograms were deconvoluted and integrated using the Automated Mass Detection and Identification System (AMDIS, NIST, USA). The quality of all software-defined peak detections and identifications was visually reviewed and manually corrected when necessary. Analytes were putatively identified by comparing mass spectral data using the NIST Mass Spectral Search Program v2.0© (Faircom Corp.; Columbia MO, USA) and cross-checking spectral matches with the experimental retention index calculated by AMDIS (RI with > 80% spectral match) of the compound. In the case of where we were unable to identify the compound, we tried to characterize its family. Some compounds remained unidentified but were still used in analysis. The final report file contained every signal contained in samples, “blanks” (intercalated with samples into batch analysis to control instrument dysfunction) and “control” samples (empty tubes and vials used to control for potential sampling contamination during storage and transport). Data processing was essentially blind as uninformative codes were given to all samples and used in all analytical steps until the final data set was obtained.

Chromatograms were aligned using the Pivot table function in Excel and alignment checked against RI values. The integrated area value of each peak (i.e., each compound, because chromatograms were characterized by several peaks where each of them represented one particular compound) was used for further processing, which involved removal of compounds consistently present in some “blanks” and “control” samples. For nest air and feather samples, areas were standardized against the internal standard (biphenyl, RI = 1439). We calculated the abundance of each compound by converting each single peak area into a percentage of the sum of all compound areas for a given sample. Therefore, all compounds from a given sample summed up to 100%. These relative abundance data were then square-root transformed to reduce the influence of the most abundant analytes in the analysis (Clarke et al. 2014). Then, we calculated Euclidean distances between every pair of samples to produce a resemblance matrix from which we conducted multivariate analyses.

### *Multivariate Analyses*

First, we looked specifically at nest air samples collected in 2014 and 2015 (N = 36 from 15 nests; see Tab. 1 for details) and to test whether each nest was characterized by a specific odor, we used the 11 nests which had at least two samples, regardless of the sampling year (N = 32; see Tab. S1 for details). We performed a between-class analysis (BCA; Dolédec and Chessel 1987) from the principal coordinate analysis (PCoA; Gower 1966) output. Then, a second BCA was performed, using only compounds shared amongst all samples from a particular nest, replacing relative abundance values with zero when it was necessary. The statistical significance of the differences between chemical cues from nests were tested with a Monte-Carlo Test on the between-groups inertia percentage using 9999 permutations.

**Table 1:** Summary of chemical compounds identified in our study, which were reported in other studies of procellariforms’ odors (feathers and/or uropygial secretion)

Compounds	Exp. RI	Family	Odor source	Occurrences in Procellariforms
Hexanal	799,1	Aldehyde	A-M-F	AP, LSP
Octane	806,3	Alkane	A-M-F	AP
Nonane	898,4	Alkane	A-M	LSP
Heptanal	901,7	Aldehyde	A-M	LSP
α-Pinene	926,1	Terpene	A-M	LSP
1-Heptanol	971,4	Alcohol	M	AP
Benzaldehyde	980,2	Benzaldehyde	A	LSP
Decane	999,2	Alkane	A-M	AP, LSP, S
Octanal	1003,6	Aldehyde	A-M	LSP
D-Limonene	1041,3	Terpene	A-M	LSP
1-Hexanol, 2-ethyl-	1042,1	Alcohol	A-F	LSP
2-Octenal, (*E*)-	1058	Aldehyde	M	AP
Undecane	1070,4	Alkane	A-M-F	LSP
Nonanal	1107,2	Aldehyde	M-F	BP*, LSP
Dodecane	1196,3	Alkane	A-M-F	LSP, S
Decanal	1204,2	Aldehyde	A-M-F	BP*, AP, LSP
2-Undecanone	1292	Ketone	M	LSP
Tridecane	1300,6	Alkane	A-M-F	LSP, S
Undecanal	1307,4	Aldehyde	A-M-F	BP*, LSP
Tetradecane	1395,7	Alkane	A-M-F	**BP**, LSP
1-Dodecanol	1491,2	Alcohol	A-F	AP
Pentadecane	1499,4	Alkane	A-M-F	**BP**, AP, LSP
Phenol, 2,4-bis(1,1-dimethylethyl)-	1555,5	Phenol	A-F	BP*
n-Tridecan-1-ol	1594,5	Alcohol	A-F	BP*
Hexadecane	1599,6	Alkane	A-M-F	BP*, LSP, S
Heptadecane	1699,5	Alkane	A-M-F	BP*, AP, LSP
Methyl tetradecanoate	1736,7	Fatty acid ester	F	S
Octadecane	1799,2	Alkane	A-F	BP*, LSP
Nonadecane	1901,7	Alkane	A-F	BP*, LSP
Hexadecanoic acid, methyl ester	1938,1	Fatty acid ester	A-F	BP*, S
Eicosane	1997,8	Alkane	A-F	BP*

Exp. RI represents the experimental retention index calculated by AMDIS.

A = nest air, M = nest material, F = feathers. AP (Antarctic Prion, *Pachyptila desolata*) in Bonadonna et al. 2007, BP (Blue Petrel, *Halobaena caerulea*) in Mardon et al. 2010, 2011, LSP (Leach’s Storm-Petrel, *Oceanodroma leucorhoa*) in Jennings and Ebeler 2020 (Cory’s Shearwater, *Calonectris borealis* and Scopoli’s Shearwater, *C. diomedea*) in Gabirot et al. 2016; Zidat et al. 2017.

*unpublished data from Mardon et al. 2011.

In a second analysis, differences between nest occupancy status (i.e., occupied or empty nest) and years of the data collection were investigated first, using PCoA to visualize the potential pattern of differences in the multivariate chemical structure among occupancy status and years. Then, permutational multivariate analysis of variance (PerMANOVA, 9999 permutations; Anderson 2001, 2017) was performed to examine whether there was a difference in chemical profiles between occupied and empty nests, as well as between 2014 and 2015. A canonical analysis of principal coordinates based on discriminant analysis (CAP; Anderson and Legendre 1999; Anderson and Willis 2003) was performed to assess the significance of class discrimination using the whole compounds. RandomForest^TM^ (RF, 10,000 trees; Breiman 2001) was used to identify the chemical compounds that were potentially important for differences between groups. Using the top compounds highlighted by RF, we conducted a second CAP to verify the power of these predictors in the discriminating between groups.

Finally, a Venn diagram was constructed to derive a global description of chemical compounds found in nest air, nest material and feather samples collected in 2014 (N = 41; see Tab. S1 for details), allowing us to highlight common and specific compounds of each odor source. We used the same methods as above to investigate for differences between odor sources. In the second step, we removed all odor source-specific compounds, creating a new dataset. We then performed the same analyses as before to disentangle the origin of chemical compounds from nest air samples.

All statistical analyses were conducted in R v. 3.6.1 (R Core Team 2022) and results were considered significant at p < 0.05. We used the “ade4” package (Dray and Dufour 2007) to perform principal coordinate analyses (*dudi.pco* function), the between-class analysis (*bca* function) and the Monte-Carlo test (*rtest* function). We also used the “vegan” package (Oksanen 2010) to carry out permutational multivariate analyses of variance (*adonis2* function) and the canonical analysis of principal coordinates based on discriminant analysis (*CAPdiscrim* function), as well as the “randomForest” package (Liaw and Wiener 2002; *randomForest* function).

## Results

Overall the same 194 compounds were detected across 60 samples of nest air, nest material and feather samples collected in 2014 and 2015 (Tab. S2). Of these, 75 (38.7%) could be putatively identified, a further seven were classified as aromatic compounds and 112 (57.7%) remained unidentified. The main chemical classes were alkanes (N = 21) followed by alkenes (N = 11), aromatic compounds (N = 7 + 3 putatively identified), ketones (N = 8), alcohols (N = 7), aldehydes (N = 7), terpenes (N = 6), fatty acid esters (N = 4) and nitrogenous compounds (N = 2) and alkynes, esters, ethers, furans, phenolic and aromatic aldehydes represented with one compound. We found that 2014 nest air samples were composed of 79 compounds. In the 2015 nest air samples we found nine additional compounds (N = 88 VOCs).

### *Nest-specific Chemical Labels*

**Fig. 2:**
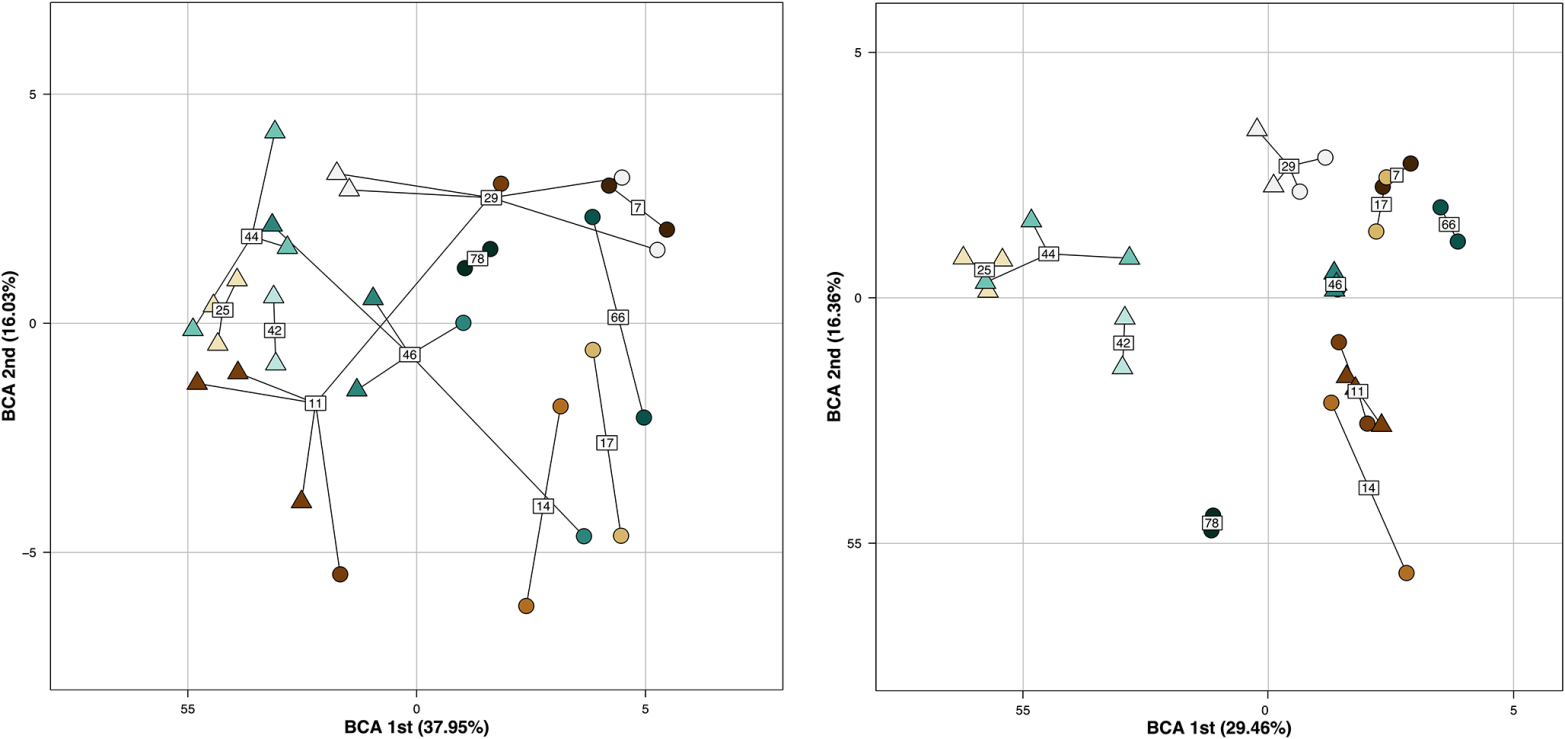
Plot of the between-class analysis (BCA) obtained by considering each nest as a class (N = 11), **a**) using the complete VOC profile (88 VOCs; Axis 1: 37.95%, Axis 2: 16.03%) and **b)** using only compounds common in all samples from a particular nest (61 VOCs; Axis 1: 29.46%, Axis 2: 16.36%). Each color represents a particular nest named using numbered labels (7, 11, 14, 17, 25, 29, 42, 44, 46, 66, 78). Dots and triangles represent samples collected in 2014 and 2015, respectively

To study whether a particular nest was defined by a specific chemical composition, we performed a between-class analysis using all compounds found in nest air samples collected in 2014 and 2015 (88 VOCs; Fig. 2a). The first two axes of the BCA captured 53.93% of the within-class variation highlighting that chemical profiles seemed to be closer within a particular nest rather than between nests. Furthermore, the Monte-Carlo test indicated an observed value of the criterion equal to 0.435 which means that 43.5% of the total inertia came from the differences between nests. This difference was highly significant (P = 0.001). This result was even more pronounced when we only kept compounds shared with all samples from a same nest (61 VOCs; Fig. 2b) where 83.7% of the total inertia came from the differences between nests (*Monte-Carlo test*; P = 0.001).

### *Nest Occupancy Status and Yearly Variation*

**Fig. 3:**
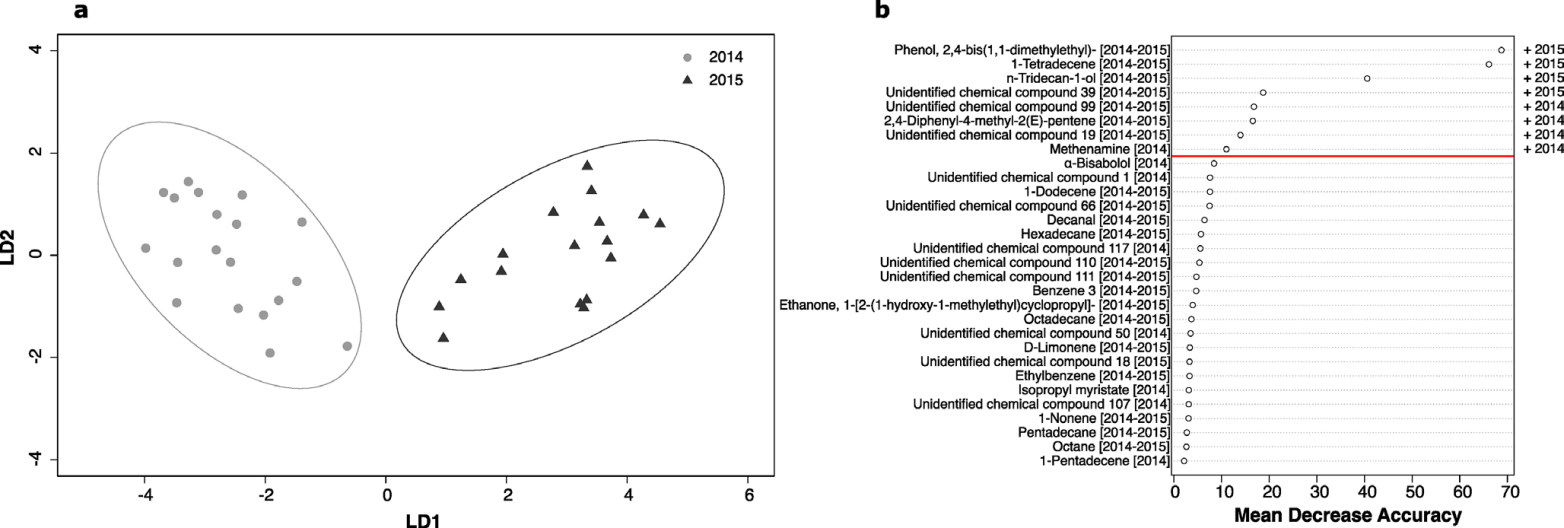
**(a)** Ordination plot from CAP of the 88 VOCs from nest air samples collected in 2014 and 2015, each ellipse represents the 95% confidence interval (SD). The percentage of correct classification in the CAP model was 100% at a confidence of P = 0.01. **(b)** Significant features identified by random forests to predict the sampling year. The compounds are ranked by the decrease in the model’s predictive accuracy from omission of the compounds in successive permutations of decision trees. Sampling years are indicated in square brackets. The “+” indicates which sampling year had the higher relative abundance of each highlighted compounds

Nest air samples collected in 2014 and 2015 (88 VOCs) showed no significant differences between occupied and empty nests (PerMANOVA, pseudo-F *F*_*1,35*_ = 0.94, P = 0.49), however the two years were clearly separated along the first axis of PCoA (Fig. S1) and significantly different in PerMANOVA (pseudo-F *F*_*1,35*_ = 8.02, P < 0.001). The two years were clearly discriminated and 100% correctly identified in CAP (Fig. 3a). Random forests highlighted eight compounds with the highest degree of discriminatory power (Fig. 3b). Despite that each year was characterized by some specific VOCs (N = 21 for 2014 and N = 9 for 2015), seven of the eight highlighted compounds were common between the two years. In particular, nest air samples collected in 2014 were mainly characterized by high relative abundances of 2,4-Diphenyl-4-methyl-2(*E*)-pentene, Methenamine and two unidentified chemical compounds. Whereas nest air samples collected in 2015 were mainly characterized by high relative abundances of Phenol, 2,4-bis(1,1-dimethylethyl)-, 1-Tetradecene, n-Tridecan-1-ol and one unidentified chemical compound (Fig. 3b). Furthermore, a repeat of CAP with these eight highlighted compounds retained a percentage of correct classification of 100%.

### *Chemical Components of Blue Petrel Nest Odor from Nest Air, Nest Material and Feathers*

**Fig. 4:**
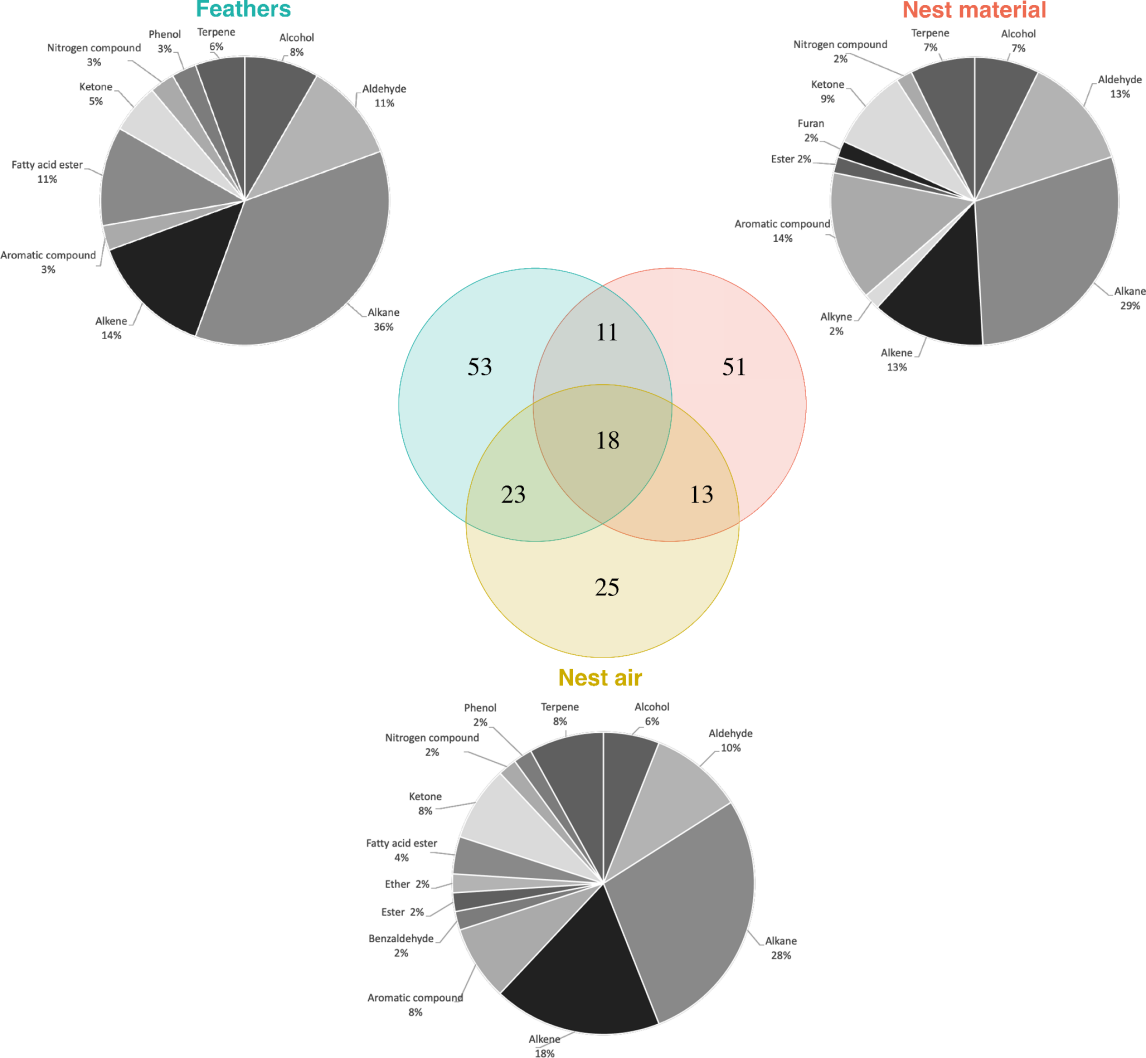
Diagrams of chemical compositions of the three odor sources collected in 2014. The colored Venn diagram illustrates the total amount of identified and unidentified compounds from each odor source. Nest air samples had N = 50 putatively identified compounds and N = 29 unidentified compounds, nest material samples N = 55 and N = 38, and feather samples N = 36 and N = 69. The pie charts correspond to the proportion of putatively identified compounds in each family class from the three odor sources

We found 194 compounds among the 43 samples collected in 2014 (i.e., N = 10 feather, N = 14 nest material and N = 19 nest air samples; see Tab. S2). Feather samples yielded the highest number of compounds (N = 105), nest air samples the fewest compounds (N = 79) and nest material samples ranked in between (N = 93, Fig. 4). The three sample sources had 18 compounds in common (Fig. 4; eight alkanes, three alkenes, three aldehydes and four unidentified compounds). Nest air samples had 13 chemical compounds in common with nest material samples (two aldehydes, two alkanes, two alkenes, two terpenes, two aromatic compounds, one ester, one ketone and one unidentified compound) and 23 chemical compounds with feather samples (three alcohols, three alkanes, two alkenes, two fatty acid esters, two ketones, two terpenes, one nitrogenous compound, one phenol derived and seven unidentified compounds), while nest material and feather samples had 11 chemical compounds in common with (one aldehyde, one alkane, one aromatic compounds and eight unidentified compounds). Each sample also showed source-specific compounds: 25 compounds from nest air (two alkenes, two aromatic compounds, one alkane, one benzaldehyde, one ether, one ketone and 17 unidentified compounds), 51 compounds from nest material (five alkanes, five aromatic compounds, four alcohols, four ketones, two alkenes, two terpenes, one aldehyde, one alkyne, one furan derived, one nitrogenous compounds and 25 unidentified compounds), and 53 compounds from feathers (one alkane, two fatty acid esters and 50 unidentified compounds).

**Fig. 5:**
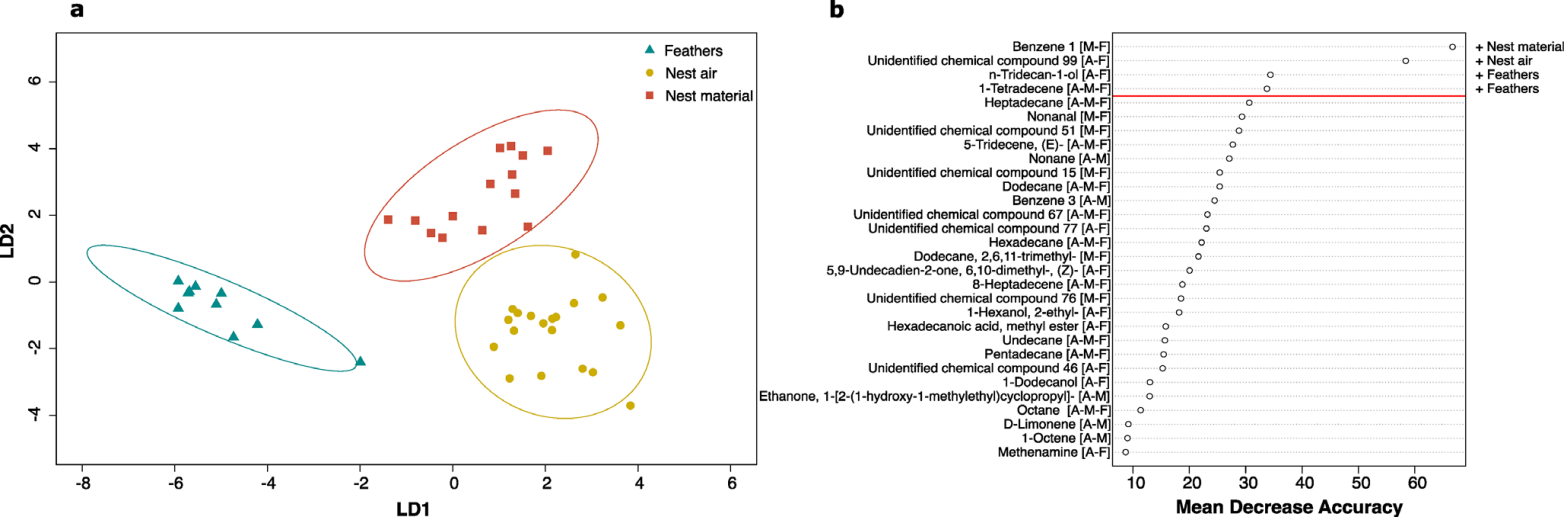
**(a)** Ordination plot from CAP of the 65 VOCs common to at least two sources, each ellipse represents the 95% confidence interval (SD). The percentage of correct classification in the CAP model was 97.67% at a confidence of P = 0.01. **(b)** Significant features identified by random forests to predict odor sources. The compounds are ranked by the decrease in the model’s predictive accuracy from omission of the compounds in successive permutations of decision trees. Original odor sources of compounds are indicated in square brackets: A = nest air, M = nest material, F = feathers. The “+” indicates which odor source had the higher relative abundance of each highlighted compounds

We found a strong significant difference in chemical composition of nest air, nest material and feather samples collected in 2014 using all the 194 compounds (PerMANOVA: pseudo-F *F*_*1,42*_ = 16.62, P < 0.001). The PCoA revealed that the two first axes captured 55.5% of the total variation and clearly separated the three odor sources (Fig. S2a). Discrimination between years was achieved in CAP with an overall 100% classification success (Fig. S2b). Random forests identified 12 compounds which had a high degree of discriminatory power between the three odor sources (highest mean decrease accuracy, Fig. S2c). Eight of these 12 compounds were specific to an odor source (N = 4, N = 3, and N = 1 for nest material, nest air and feather samples respectively). Compounds that have been found in more than one source are likely to have originated from only one source and have been transferred to others through close contact with the source. Therefore, we focused on 65 compounds shared by at least two sources to determine the original source of each compound (see Fig. 4). We still found a significant chemical variation between the three odor sources (PerMANOVA: pseudo-F *F*_*1,42*_ = 12.01, P < 0.001; see Fig. S3 for ordination plot from PCoA) and 97.6% correct classification in the CAP model (one feather sample was misclassified; Fig. 5a). Random forests identified four compounds with the highest degree of discriminatory power (Fig. 5b) and CAP based on these compounds resulted in 97.7% (one nest material sample was misclassified). In particular, three of these compounds (n-Tridecan-1-ol, 1-Tetradecene and one unidentified chemical compound) were shared with nest air and feather samples with n-Tridecan-1-ol and 1-Tetradecene relatively more abundant in feather samples than nest air samples (Fig. 5b).

## Discussion

In this study, we investigated the chemical composition of blue petrel nest odors, a species that is assumed to use its excellent sense of smell in short-range homing and nest recognition. Specifically, we used an innovative method to capture, extract, and identify chemical compounds from nest odor of blue petrels during the breeding season.

We used in situ sampling of nest air onto TD tubes to capture, analyze, and identify chemical compounds from blue petrel nest odors during the breeding season. To better understand the chemical composition of these nest air samples, we compared it to nest material and feather samples. We found that nest air odors were partially formed by compounds from nest material and owners’ feathers. As well as, bird-derived compounds seemed to have a greater contribution to the chemical composition of nest air, which probably contributes to the nest specific chemical label we highlighted. We also found that, despite a chemical differentiation between years, nest air odors seemed stable over the breeding season. These findings, associated with the previous behavioral experiments, strongly suggest that the scent emanating from blue petrel burrows may provide information to facilitate nest recognition.

As expected, but never described, our results demonstrate the presence of a nest-specific chemical mixture which might be important to find the location of nests and recognize them when blue petrels arrive at their colony (Bonadonna et al. 2004). In fact, in choice experiments, birds, removed few minutes from their own burrow before the experimentation was done, showed preference for their own nest odors over a conspecific nest odors (Bonadonna et al. 2004). We highlighted that the main component of the scent emanating from burrows originates directly or indirectly from birds. As well, we know that blue petrels have individual odors (Mardon et al. 2010, 2011). They can also recognize and discriminate individual odors, in particular their partners’ odor (Mardon and Bonadonna 2009). To conclude, these results reinforce the idea that nests’ odor is most likely a mixture of nest material and both partners’ odors that the birds can learn to recognize.

To allow nest recognition over the breeding season year after year, nest air odors should be persistent over time. Indeed, we found no difference in chemical composition between occupied nests and empty nests within the breeding season, suggesting that nest odors were persistent over time. The blue petrel, as other Procellariform species, is known for its strong odor. This is why the cotton bags used for transporting them are often used as odor sources in behavioral experiments, as they are permeated by birds’ scent (e.g., Bonadonna and Nevitt 2004). In the same way, it is therefore very likely that birds’ odor also permeates burrows. Indeed, it was suggested that birds might unintentionally rub themselves on the walls of the burrow because the blue petrel nest cavity is quite small. Thus, these birds might depose their odor on plants, roots, soil and everything in the nest (Bonadonna et al. 2004). In particular, the egg might be the main material smelling of the incubating bird odor. Indeed, although birds readily accept an artificial egg exchange without any rejection problems (F. Bonadonna personal observation), Leclaire et al. (2017) showed that females could recognize their own egg solely by chemical cues and suggested that this egg-odor recognition could be a by-product of burrow-odor recognition.

We identified eight chemical compounds that were primarily responsible for interannual differences in chemical profiles from nest air odors. Food availability is likely to vary temporally and dietary shifts can impact chemical profiles (e.g., Apandi and Edwards 1964; Reneerkens et al. 2007; Thomas et al. 2010; Grieves et al. 2020). Depending on the availability of their prey sources, the composition of blue petrels’ diet could differ across years, leading to shifts in their chemical profiles. Furthermore, we found several compounds that were presumably plant-derived (terpenes) and may vary between the two years. Moreover, annual differences in the chemical composition of nest air odors could also be due to (i) a variation in the plant composition around nests and the plants use to make the nest and/or (ii) plant-derived compounds that birds may involuntarily catch on their feathers when they return to their nest. Finally, we identified a few compounds previously found in Antarctic prion’s feathers (Bonadonna et al. 2007). Blue petrels and Antarctic prions are known to nest in sympatry on the majority of Kerguelen Islands (Cherel et al. 2002a, b) and nest squatting was observed on islands such as Verte Island where interspecific competition for nest is high (Bonadonna and Mardon 2013). The interannual differences in chemical profiles from nest air odors may also be due to the degree of squatting by other species or other blue petrels. However, this interannual variation in the chemical composition of nest air odor is presumably lower than the strong variation among different nests we found. Furthermore, this chemical variation seems not to affect the nest recognition year after year, which suggests a consistent nest chemical base that persists over the years.

Procellariforms are known for the strong musky odor of their plumage, which is impossible to miss when we handle these birds in the field as well as “sticks” or objects that touch the birds’ feathers (personal observation). These compounds produced by birds constitute a part of the nest air odor and our results support that. In our study, nest air odor was a blend of 23 compounds shared with feather samples, 13 shared with nest material samples and 25 specific compounds. It’s very likely that these shared compounds took their sources from feathers and nest material. Focusing on compounds shared between nest air and feather samples, for example, we noticed that eight compounds that were previously identified in birds’ odors (see Tab. 2 for references). In particular, six of the eight (Phenol, 2,4-bis(1,1-dimethylethyl)-, n-Tridecan-1-ol, Octadecane, Nonadecane, Hexadecanoic acid, methyl ester and Eicosane) had previously been reported in blue petrels (Mardon et al. 2010, 2011) and most certainly originated from the birds’ uropygial secretion. Furthermore, we also found two terpenes (α-Pinene and D-Limonene), known as plant-derived volatiles, in nest material samples and the majority of nest air samples as well. This result is not surprising because basic nests are made up of plants and roots, mainly dead branches of *Acaena magellanica* (personal observation). This suggests that chemical transfers from nest material to nest air may occur. Another interesting feature are compounds shared between nest material and feather samples. We know that nest material consists of owners’ feathers, so a part of its chemical composition could come indirectly from birds. Indeed, 11 compounds were shared between nest material and feather samples (Tab. 2) including nonanal which was previously found in the blue petrels (Mardon et al. 2010, 2011). Furthermore, 11 of the 18 compounds common to all three sources were previously reported in seabirds (Tab. 2), specifically two aldehydes (Decanal, Undecanal) and four alkanes (Tetradecane, Pentadecane, Hexadecane, Heptadecane) were found in blue petrels (Mardon et al. 2010, 2011). These results suggest that nest air odor is a mixture of birds’ odors and nest material coming mainly from birds, either directly from incubating birds or indirectly from dropped feathers inside nests.

## Conclusion

To best of our knowledge, we present here the first description of chemical signatures of burrows of blue petrels and show that these signatures predominantly consist of chemical compounds released by birds over years, supporting their role in homing behavior and nest recognition. Until now many works discussed the likely role of olfaction in aiding these birds to return each year to reproduce in their own burrow with their partner but never described the chemistry (review in Bonadonna 2009; Bonadonna and Mardon 2013). In addition, we describe a reliable and repeatable method for *in situ* capture and subsequent identification of volatile organic compounds emitted by the burrows of breeding birds.

### Electronic Supplementary Material

Below is the link to the electronic supplementary material.

Supplementary Material 1

Supplementary Material 2
