# Supplementary material for: Homing and Nest Recognition in Nocturnal Blue Petrels: What Scent May Attract Birds to their Burrows?
Source: J Chem Ecol. 2023 May 26;49(7-8):384–96. doi: 10.1007/s10886-023-01424-3 (PMC10611862; doi:10.1007/s10886-023-01424-3)
Supplement: Supplementary file 1 — Supplementary Material 1 [file 10886_2023_1424_MOESM1_ESM.docx]

**Table S1**:

SUMMARY OF THE ALL SAMPLES USED FOR ANALYSES PER NEST, ODOR SOURCE AND SAMPLING YEAR

*Note: Breeding birds are named by their ring (GX)*

| Nest | Sample | **2014** | | | **2015** |
| --- | --- | --- | --- | --- | --- |
|  |  | Feather | Nest material | Nest air | Nest air |
| **3** | GX24042 |  |  |  | x |
| **7** | GX26139 | x |  | x |  |
|  | GX24116 |  | x | x |  |
| **11** | GX22006 |  | x | x | x |
|  | GX26098 |  |  |  | x |
|  | empty nest |  |  | x | x |
| **14** | GX24028 |  |  | x |  |
|  | empty nest |  |  | x |  |
| **17** | GX22010 | x | x | x |  |
|  | empty nest |  | x | x |  |
| **25** | GX24123 |  |  |  | x |
|  | GX29510 |  |  |  | x |
|  | empty nest |  |  |  | x |
| **29** | GX24186 | x | x | x |  |
|  | GX27292 |  | x | x | x |
|  | empty nest |  |  |  | x |
| **34** | GX27339 |  |  | x |  |
| **42** | GX26127 |  |  |  | x |
|  | GX29518 |  |  |  | x |
| **44** | GX26019 | x |  |  | x |
|  | GX24159 |  |  |  | x |
|  | empty nest |  |  |  | x |
| **46** | GX22430 | x | x | x | x |
|  | GX26045 | x | x | x | x |
|  | empty nest |  |  |  | x |
| **47** | GX24027 | x |  |  |  |
| **58** | GX24130 | x | x | x |  |
|  | GX24183 |  | x |  |  |
| **66** | GX24194 |  | x | x |  |
|  | GX26144 |  |  | x |  |
|  | empty nest |  | x |  |  |
| **75** | GX24149 | x | x |  |  |
|  | GX26122 | x | x | x |  |
| **78** | GX27335 |  |  | x |  |
|  | GX27351 |  |  | x |  |
|  | | | | | |


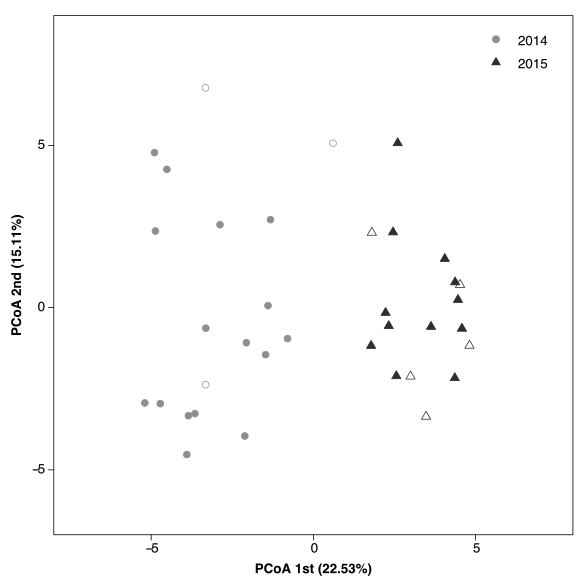


**Fig. S1** Ordination plot from PCoA based on the 88 VOCs from nest air samples, sampled in 2014 and 2015. The two principal axes captured 37.64% of the total variation (Axis 1: 22.53%, Axis 2: 15.11%). Dots and triangles represent samples collected in 2014 and 2015, respectively. Empty symbols represent samples from empty nests, whereas solid symbols represent samples from occupied nests (i.e., with an incubating adult inside)

**Fig. S2** **a)** Ordination plot from PCoA based on the all 194 VOCs from the three odor sources collected in 2014. The two principal axes captured 47.3% of the total variation (Axis 1: 26.63%, Axis 2: 20.67%). Colored symbols represent the odor sources (blue triangles for feather, red squares for nest material and yellow circles for nest air samples). **b)** Ordination plot from CAP of the 194 VOCs from the three odor sources where each ellipse represents the 95% confidence interval (SD). The percentage of correct classification in the CAP model was 100% at a confidence of P = 0.01. **c)** Significant features identified by random forests to predict odor sources. The compounds are ranked by the decrease in the model’s predictive accuracy from omission of the feature in successive permutations of decision trees. Original odor sources of compounds are indicated in square bracket

**Fig. S3** Ordination plot from PCoA based on the 65 common VOCs (see **Fig. 1**). The two principal axes captured 44.07% of the total variation (Axis 1: 27.80%, Axis 2: 16.27%). Colored symbols represent the odor sources (blue triangles for feather, red squares for nest material and yellow circles for nest air samples)
